# Development of an Indian Food Composition Database

**DOI:** 10.1016/j.cdnut.2024.103790

**Published:** 2024-06-13

**Authors:** Aswathy Vijayakumar, Hima Bindu Dubasi, Ananya Awasthi, Lindsay M Jaacks

**Affiliations:** 1 Anuvaad Solutions, New Delhi, India; 2 Global Academy of Agriculture and Food Systems, University of Edinburgh, Midlothian, United Kingdom

**Keywords:** food composition tables, nutrient data, Indian Nutrient Databank, raw food items, composite dishes

## Abstract

An open-access and comprehensive nutrient database is not available in India. Our objective was to develop an open-access Indian Nutrient Databank (INDB). The development of the INDB consisted of 2 stages: creating a database of the nutrient composition data of individual food items (*n* = 1095) and a database of commonly consumed recipes (*n* = 1014). The stage 1 database was primarily derived from the Indian Council of Medical Research-National Institute of Nutrition’s Indian Food Composition Table (ICMR-NIN IFCT) from 2017, with gaps filled using the ICMR-NIN IFCT 2004 and nutrient databases from the United Kingdom and United States. The stage 2 database included information on the amounts of each ingredient used in each recipe, matched to a comparable item in the database from stage 1. This unique open-access resource can be used by researchers, the government, and the private and third sectors to derive nutrient intakes in India to better inform interventions and policies to address malnutrition.

## Introduction

Food composition databases or food composition tables (FCTs) play an important role in diet assessment by allowing users to convert foods consumed by individuals into estimates of nutrient intake [1,2]. This is a required step in monitoring population nutrient intake relative to recommended intake levels and dietary guidelines [2,3]. Thus, comprehensive, context-specific FCTs are critical to advance nutritional research and policy [3].

In India, the nutrient composition of hundreds of raw food items has been quantified by laboratories at the Indian Council of Medical Research-National Institute of Nutrition (ICMR-NIN) [4]. The latest version of the ICMR-NIN Indian Food Composition Tables (IFCTs) is available from the year 2017. ICMR-NIN used a “key foods approach” to prioritize food items for extracting the nutrient composition, meaning that the food items that together contribute to 75% of population intake of energy, fat, protein, and 8 micronutrients were identified and included in the IFCT. The IFCT 2017 includes nutrient composition data for 528 raw food items. For example, uncooked rice, wheat flour, and tomatoes. However, it does not include data for composite dishes, i.e., chapattis or curries.

India is known for its rich dietary diversity and heterogeneity of composite dishes. However, the nutrient composition of composite Indian dishes is limited. The nutrient composition of specific dishes, such as dishes cooked in Himachal Pradesh using sesame seeds or cereals and pulses-based recipes from Assam, have been done using proximate and micronutrient composition analysis [[5], [6], [7], [8]]. Websites such as RecipeDB and mobile applications such as HealthifyMe have data on nutrients in Indian recipes [9]. However, these resources lack documentation of their methodology, and the databases underlying them are not open-access.

The objective of this study was to develop an open-access Indian Nutrient Databank (INDB) consisting of both raw food items and standard Indian recipes. The INDB can be used by researchers, the government, and the private and third sectors to derive the nutrient intakes of individuals and populations.

## Methods

The development of the INDB consisted of 2 stages (Supplemental Figure S1). First, a database of the nutrient composition data of raw food items was created from the IFCT 2017 and 2004 [4]. When foods were duplicated in the 2004 and 2017 versions, the most recent (2017) was used. The nutrient values of a total of 528 raw food items were available in the IFCT 2017, and nutrient values of another 369 raw food items were obtained from the IFCT 2004. In IFCT 2017, energy values were available in kilojoules (kJ), and in the 2004 version, energy values were available in kilocalories (kcal). Conversion factors from the FAO were used: 1 kJ = 0.239 kcal and 1 kcal = 4.184 kJ [10]. In IFCT 2017 and 2004, the values of some of the nutrients, such as carbohydrates, fiber, and biotin, were missing for several food items, especially protein foods (e.g., eggs, meat, and fish). These values were taken from the United Kingdom FCTs, which are freely available from Public Health England and include a large range of Indian foods due to the popularity of Indian cuisine in the United Kingdom [11]. Thus, in the final combined raw food items, the sources of information have been identified as primary and secondary sources, where the IFCT 2017 or IFCT 2004 is the primary source, and the United Kingdom FCT is the secondary source.

Second, we created a database of commonly consumed recipes across the Indian population. Most recipes were taken from 2 manuals: “Basic Food Preparation, 4^th^ Edition” (611 recipes) and “The Art of Science of Cooking, 5^th^ Edition” (513 recipes) [12,13]. Both of these manuals were developed by researchers at The University of Delhi as a reference for food and nutrition students across India. Some recipes were found in both books. The duplicate recipe information from the “Basic Food Preparation, 4^th^ Edition” was deleted from the final recipe database (*n* = 258). As these manuals are >10 y old, we supplemented these recipes with 148 recipes from popular Indian food blogs. Thus, the total number of recipes in the database was 1014. Unique recipe codes were assigned to each of the recipes. Additionally, the recipe names were updated to include the English name and local or common names of the recipes.

The recipe database was created as a Microsoft Excel file consisting of information on the amounts of each ingredient used in each recipe, including spices, the total weight of the recipe (g), the number of servings in the recipe, and the serving size. The amounts of the raw ingredients were entered as grams, milliliters, tablespoons, teaspoons, or sprigs. Each ingredient was matched to a comparable food in the FCT. We prioritized matching to the IFCT 2017 and IFCT 2004 when nutrient composition data were available for a given ingredient. If the ingredient was not in either version of the IFCT, we instead matched it to the United Kingdom FCT (*n* = 144 ingredients). If the ingredient was also not in the United Kingdom FCT, it was matched to the United States Department of Agriculture (USDA) food composition database (*n* = 54 ingredients) [11,14]. Serving size information was missing for recipes belonging to the food category “pickles and preserves” and “weaning and low-cost nutritious foods.”

In a sensitivity analysis to account for nutrient loss that occurs while cooking, data from the USDA Table of Nutrient Retention Factors, Release 6 (2007) were used [15]. The USDA Table of Nutrient Retention Factors provides data on nutrient retention factors for a range of cooking and preparation methods, such as baking, boiling, and reheating [15]. A single nutrient retention factor was applied for each raw food available in the USDA Table of Retention Factors based on the most common cooking method for that raw food in the Indian context. For example, legumes such as “kidney beans” were associated with the nutrient retention values for “legumes, cooked 15-20 minutes, boiled and baked.” The following nutrients had nutrient retention values available in the USDA Table of Nutrient Retention Factors and were included in the INDB: calcium, iron, magnesium, phosphorus, potassium, sodium, zinc, copper, vitamin C, thiamine, riboflavin, niacin, folic acid, vitamin B6, folate, vitamin A, and carotenoids. In the current Indian recipe databank, only 42.62% of the ingredients in the recipes were matched with a nutrient retention factor value. The nutrient retention factor values were multiplied by the nutrient values of the raw food items to account for the final nutrient values after cooking. Wilcoxon signed rank tests were used to test for differences in nutrient values without and with accounting for nutrient retention factors.

The nutrient content of all ingredients in a recipe was summed to obtain the total nutrient content per 100 g of the recipe and per serving of the recipe. The database consists of nutritional values for energy (kJ), energy (kcal), carbohydrates (g), protein (g), fat (g), free sugars (g), fiber (g), saturated fatty acid (mg), monounsaturated fatty acid (mg), polyunsaturated fatty acid (mg), cholesterol (mg), calcium (mg), phosphorus (mg), magnesium (mg), sodium (mg), potassium (mg), iron (mg), copper (mg), selenium (μg), chromium (mg), manganese (mg), molybdenum (mg), zinc (mg), vitamin A (μg), vitamin E (mg), vitamin D2 (μg), vitamin D3 (μg), vitamin K1 (μg), vitamin K2 (μg), folate (μg), thiamine (mg), riboflavin (mg), niacin (mg), pantothenic acid (mg), vitamin B6 (mg), biotin (μg), folic acid (μg), vitamin C (mg), and carotenoids (μg).

All calculations were done using Stata version 17, and figures were generated using Rstudio version 2023.03.1+446.

## Results

**FIGURE 1:**
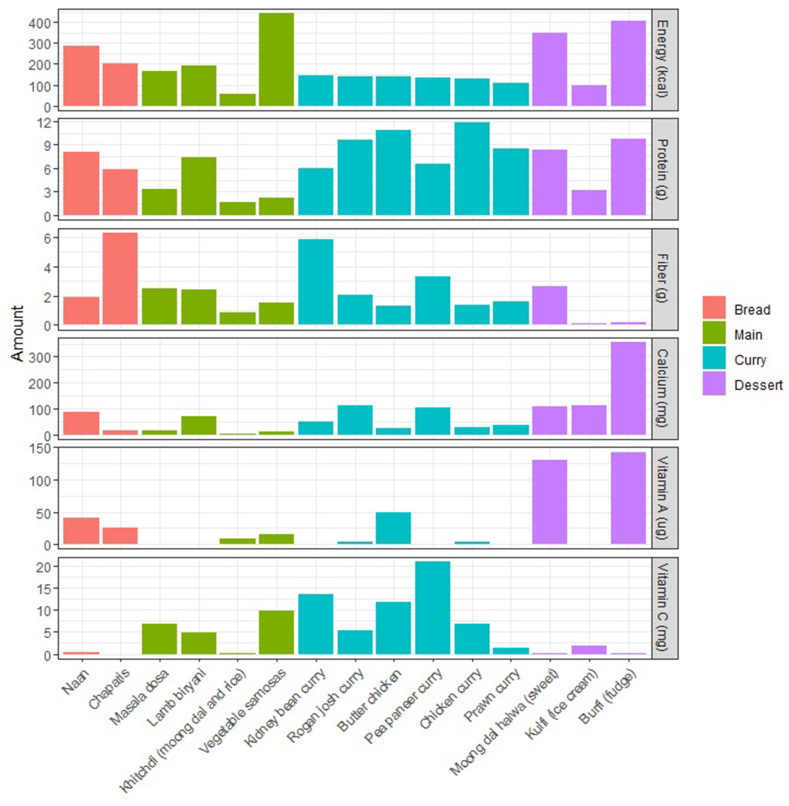
The nutrient contents [energy (kcal), protein (g), fiber (g), calcium (mg), vitamin A (μg), and vitamin C (mg), not accounting for nutrient retention factors] of commonly consumed composite dishes. The dishes belong to 4 categories, “bread”, “main”, “curry,” and “dessert”.

A total of 1095 raw food items and 1014 recipes were included in the final INDB. The nutrient content of commonly consumed composite dishes varied considerably (Figure 1). For example, the energy content of 100 g vegetable samosas was 443 kcal compared with 57 kcal for 100 g khichdi (a dish made from rice and lentils). Similarly large variability was seen for minerals and vitamins.

**FIGURE 2:**
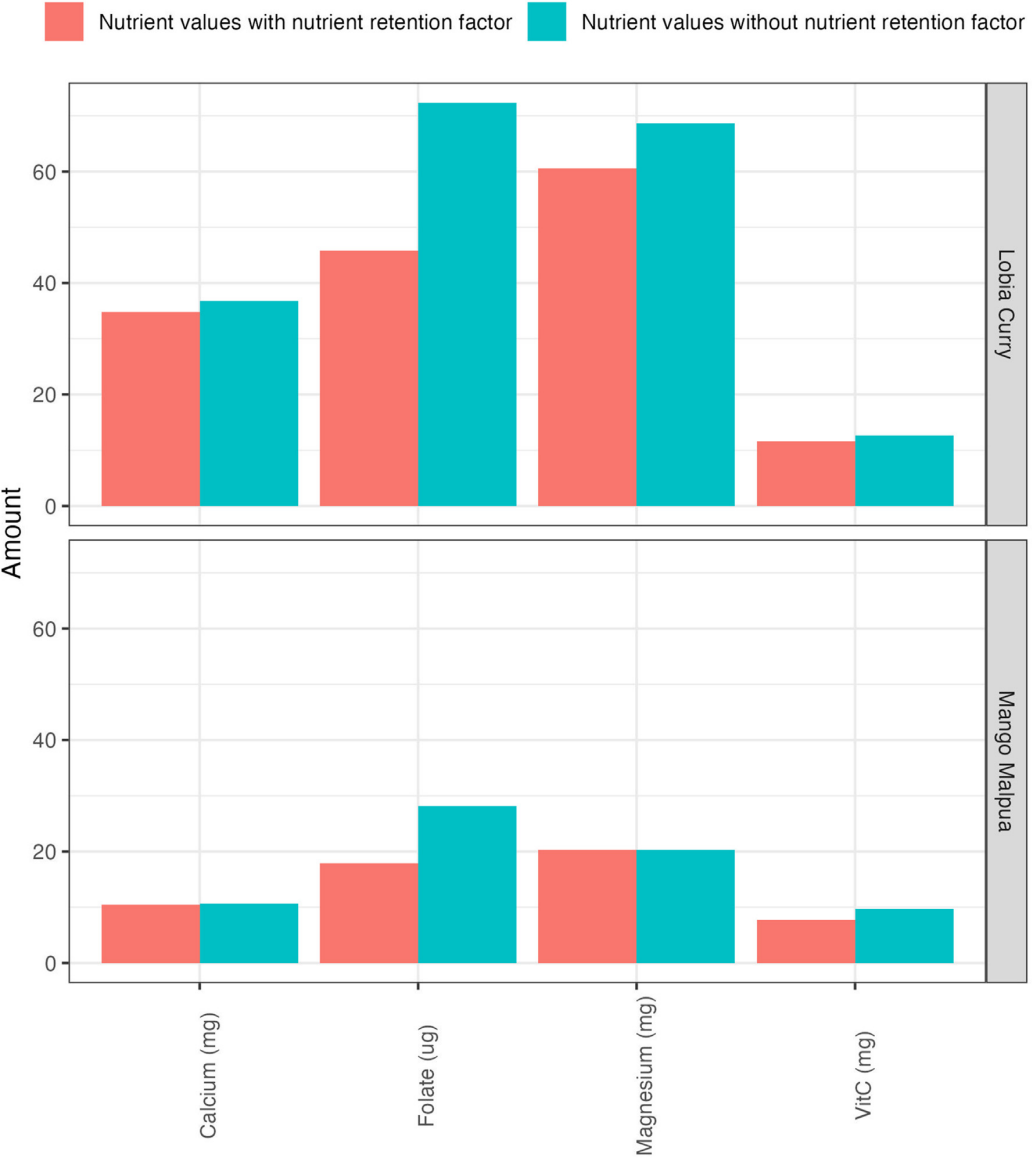
Change in nutrient composition for calcium (mg), magnesium (mg), vitamin C (mg), and folate (μg) with and without accounting for nutrient retention factors for 2 composite dishes, “Lobia Curry” (a pulse-based curry), and “Mango Malpua” (a dessert).

Nutrient retention was high for most nutrients, with the highest for calcium and zinc (75% – 100%) and the lowest for vitamin C (20% – 100%) (Supplemental Table S1). The mean difference in nutrient content without and with accounting for nutrient retention factors ranged from a decrease of 0.001mg for copper (median copper content was 0.08 mg per 100 g both without and with accounting for nutrient retention factors) to a decrease of 5.15 mg for potassium (median potassium content was 153 mg per 100 g without and 149 mg per 100 g with accounting for nutrient retention factors). Comparing median nutrient values without and with accounting for nutrient retention factors, all P values were < 0.01, indicating that the difference was statistically significant. However, the size of the difference was small, for example, the impact of accounting for nutrient retention factors on calcium, magnesium, vitamin C, and folate is shown in Figure 2.

The nutrient values for comparable composite dishes in the INDB differed from those in the United Kingdom FCT nonsystematically, meaning the INDB was higher for some nutrients and some composite dishes, whereas it was lower for others (Supplemental Figure S2).

## Discussion

A comprehensive FCT is necessary for accurately estimating the dietary intake of a population. Despite the heterogeneity of composite dishes, there is no comprehensive open-source database with a well-documented methodology on typical recipes commonly consumed by the Indian population. Thus, the objective of this study was to develop a comprehensive open-access INDB with the nutrient composition of raw food items and typical composite dishes consumed by the Indian population. This article described the methodology used in the development of the INDB, which includes 1095 raw food items and 1014 recipes.

Cooking can result in the loss of nutrients [16,17]. We found that median nutrient values were statistically significantly lower when nutrient retention factors were accounted for, but the magnitude of differences was small. The highest nutrient losses occurred for vitamin C, potassium, and phosphorus. It is possible further nutrient losses occur that were not accounted for by using the USDA Table of Nutrient Retention Factors, considering some raw foods included in the INDB were not available in the USDA database and cooking practices may differ. Analyzing cooked samples would be more accurate [18] but is cost-prohibitive, especially in contexts such as India, with a huge diversity of common recipes.

The ICMR-NIN used the “key foods approach” to prioritize the raw food items for extracting the nutrient composition. The raw food items that contribute to 75% of the total fat, protein, energy, iron, calcium, phosphorus, vitamin A, vitamin B1, vitamin B2, vitamin B3, and vitamin C were identified for extracting the nutrient composition [4]. The 2017 IFCT data includes key foods averaged from 6 geographical regions across India and increasing the food items in the “key foods approach” will also increase the geographical diversity of the raw food items included. Evidence from other studies indicates that the nutrient content of foods is going down over time and that nutrient content varies widely depending on where the food was produced [19,20]. The INDB does not take this into account and thus may be misestimating nutrient content. Values differed to some extent from comparable composite dishes in the United Kingdom FCT. These differences could be due to a combination of factors, including true underlying differences in the nutrient composition of ingredients, differences in recipes for the same dish in the United Kingdom compared with India, and differences in the laboratory methodology for assessing nutrients.

One of the limitations of the INDB is the lack of nutrient data on packaged food items. In a longitudinal, representative sample of urban India (Kantar Worldpanel, 78,320 households), purchases of sweet and salty snacks and other processed foods (largely noodles) have increased over time but remain relatively low at the population level compared with countries such as the United States and United Kingdom [21,22]. For example, the average annual purchase of salty snacks was just 1.1 kg per household member in India in 2017 compared with 9.5 kg in the United States in 2015 [21]. An updated INDB with accurate nutrient information on packaged food items is needed to accurately estimate the dietary intake of the Indian population as the consumption of processed foods continues to grow. Other next steps include correction for loss or gain in weight while cooking, generally referred to as yield factor, and expanding the number of recipes to include more regional dishes.

In conclusion, the INDB is a unique open-access resource that can be used by researchers, the government, and the private and third sectors to derive nutrient intakes in India to better inform interventions and policies to address malnutrition.

## Author contributions

The authors’ responsibilities were as follows – LMJ, AA, AV: contributed to the research design; AV, LMJ, HBD: directly assessed and verified the underlying data; AV: conducted the statistical analyses with supervision from LMJ; AV: wrote the first draft of the manuscript; and all authors: read and approved the final manuscript.

## Conflict of interest

The authors report no conflicts of interest.

## Funding

This research was funded by The Bill and Melinda Gates Foundation. For the purpose of open-access, the author has applied a Creative Commons Attribution (CC BY) license to any Author Accepted Manuscript version arising from this submission.

## Data availability

All analysis codes and files are publicly and freely available on GitHub: https://github.com/lindsayjaacks/Indian-Nutrient-Databank-INDB-.
