# Supplementary material for: Development of an Indian Food Composition Database
Source: Curr Dev Nutr. 2024 Jun 13;8(7):103790. doi: 10.1016/j.cdnut.2024.103790 (PMC11277795; doi:10.1016/j.cdnut.2024.103790)
Supplement: Multimedia component 1 [file mmc1.docx]

**Development of an Indian Food Composition Database**

Aswathy Vijayakumar,^1*^ Hima Bindu Dubasi,^1^ Ananya Awasthi,^1^ Lindsay M Jaacks^2^

^1^ Anuvaad Solutions, New Delhi, India

^2^ Global Academy of Agriculture and Food Systems, University of Edinburgh, Midlothian, UK

*Correspondence: Dr Aswathy Vijayakumar, [aswathy@anuvaad.org.in](mailto:aswathy@anuvaad.org.in)


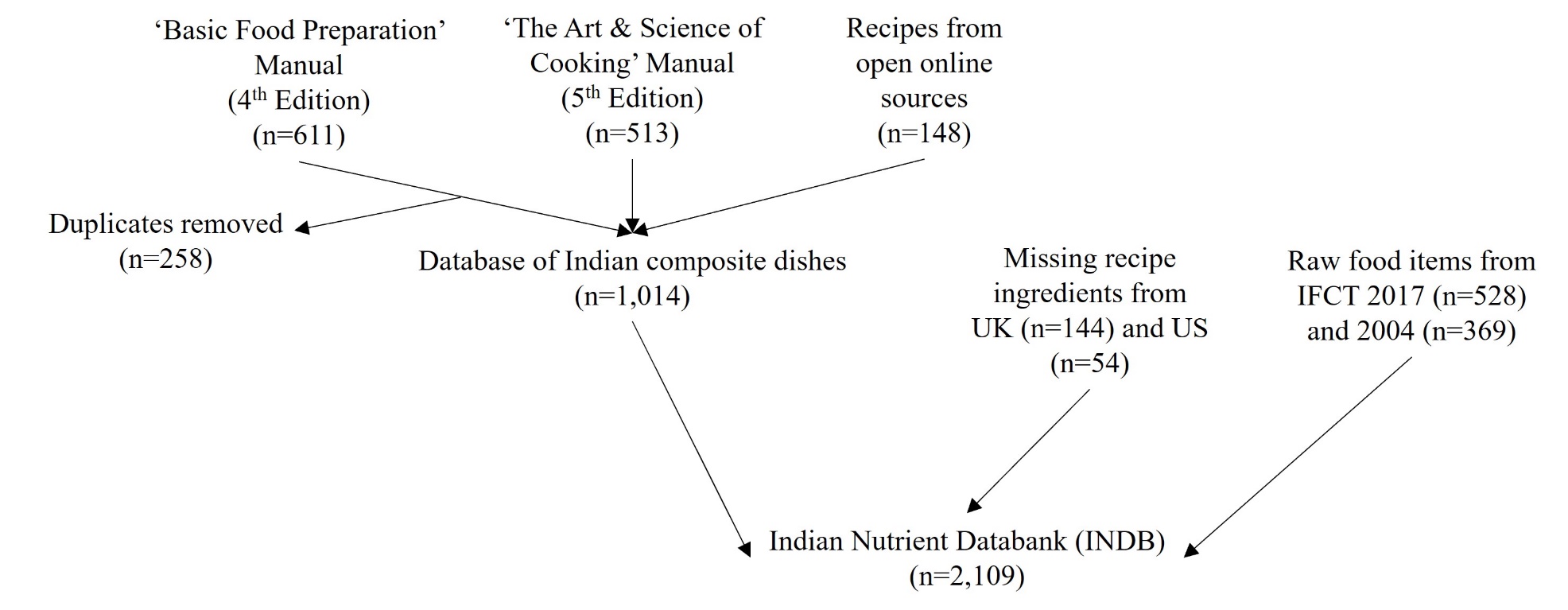


**Supplemental Figure S1.** Sources of data for the Indian Nutrient Databank.


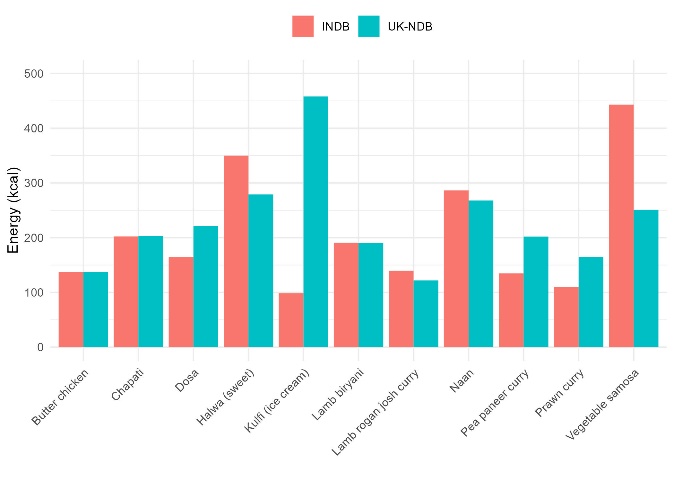

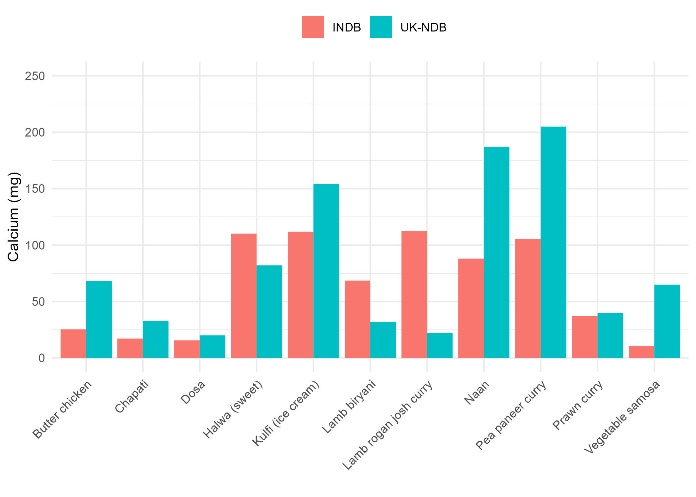


**Supplemental Figure S2.** The distribution of energy (kcal) and calcium (mg), not accounting for nutrient retention factors, for 11 comparable composite dishes in the Indian Nutrient Databank (INDB) and the UK food composition table.

**Supplemental Table T1. Summary statistics of nutrient content per 100g with and without accounting for nutrient retention factors**

|  | **Without retention factor** | **With retention factor** |  |  |
| --- | --- | --- | --- | --- |
|  | **Median (IQR)** | **Median (IQR)** | **Mean difference** | **Range retention factor (%)*** |
| Calcium (mg) | 40.79 (60.18) | 40.30 (60.48) | -0.17 | 75-100 |
| Iron (mg) | 0.83 (0.83) | 0.82 (0.82) | -0.01 | 60-100 |
| Magnesium (mg) | 18.90 (18.55) | 18.77 (18.06) | -0.37 | 60-100 |
| Phosphorus (mg) | 75.58 (74.61) | 74.02 (75.12) | -1.65 | 65-100 |
| Potassium (mg) | 153.16 (154.95) | 148.94 (155.19) | -5.15 | 30-100 |
| Sodium (mg) | 101.05 (154.84) | 101.05 (154.05) | -0.13 | 45-100 |
| Zinc (mg) | 0.46 (0.45) | 0.46 (0.45) | -0.005 | 75-100 |
| Copper (mg) | 0.08 (0.08) | 0.08 (0.08) | -0.001 | 55-100 |
| Vitamin C (mg) | 3.11 (7.99) | 2.53 (6.83) | -1.19 | 20-100 |
| Thiamine (mg) | 0.05 (0.06) | 0.04 (0.04) | -0.01 | 30-100 |
| Riboflavin (mg) | 0.06 (0.06) | 0.05 (0.07) | -0.002 | 55-100 |
| Niacin (mg) | 0.37 (0.6) | 0.35 (0.55) | -0.04 | 45-100 |
| Folic acid (µg) | 15.13 (16.69) | 11.96 (13.16) | -4.27 | 25-100 |
| Vitamin B6 (mg) | 0.07 (0.07) | 0.06 (0.07) | -0.008 | 35-100 |
| Folate (µg) | 15.13 (16.7) | 11.96 (13.16) | -4.27 | 25-100 |
| Vitamin A (µg) | 8.10 (75.0) | 7.94 (74.63) | -0.04 | 50-100 |
| Carotenoids (µg) | 256. 62 (852.57) | 245.13 (804.24) | -39.44 | 50-100 |

*USDA nutrient retention factor values, Release 6 (2007).
